# Neural correlates of emotional prosody in Parkinson’s disease: A systematic review

**DOI:** 10.3758/s13415-025-01379-w

**Published:** 2026-01-08

**Authors:** Sharon Mara Luciano, Francesco Panico, Rosalia De Biase, Laura Catalano, Laura Sagliano, Luigi Trojano

**Affiliations:** Department of Psychology, University of Campania “Luigi Vanvitelli”, Viale Ellittico 31, 81100 Caserta, Italy

**Keywords:** Parkinson disease, Emotional prosody, Neuroimaging, Electroencephalography, Brain stimulation

## Abstract

Parkinson’s disease (PD) is a chronic neurodegenerative disorder involving motor, cognitive and affective symptoms. Processing of emotional prosody, namely the modulation of pitch, volume, and frequency in speech conveying emotional nuances, seems to be affected in PD, but its neural mechanisms have not been fully comprehended. To address this issue, the present systematic review searched for the studies which investigated the neural correlates of emotional prosody in PD patients by means of electrophysiological, neuroimaging, and brain stimulation techniques. Studies on deep brain stimulation of the subthalamic nucleus provided conflicting information on the alterations in emotional prosody perception and expression, with some inconsistency about lateralization. Brain recording and imaging techniques revealed associations of perception and expression deficits of emotional prosody with striatal, frontal, and limbic dysfunction. No study adopted non-invasive brain stimulation to assess emotional prosody processing. Future research should incorporate these techniques in experimental settings and use standardised emotional prosody tasks in patients with PD and in control healthy individuals. Clarifying the neural correlates of emotional prosody may enhance understanding of social communication deficits and could be informative for future therapeutic approaches to improve quality of life in patients with PD.

**Supplementary Information:**

The online version contains supplementary material available at 10.3758/s13415-025-01379-w.

## Introduction

Parkinson’s disease (PD) is a chronic neurodegenerative disorder, primarily resulting from dopamine depletion in the nigrostriatal pathway (Péron et al., 2012). PD is characterized by motor symptoms such as tremors, rigidity, and bradykinesia (Cabreira & Massano, 2019). However, an increasing number of studies reported emotional and affective symptoms in PD, which seem to be mainly related to the progressive loss of dopaminergic neurons in the mesolimbic pathways projecting to the basal ganglia and the frontal cortex (Li et al., 2019; Oosterwijk et al., 2018; Ramesh & Arachchige, 2023). Emotion processing is indeed impaired in PD (Péron et al., 2012; Vicente et al., 2011), as patients show difficulties in recognizing emotions expressed by the face (Péron et al., 2013) or voice (Argaud et al., 2018). For instance, patients with PD perform worse than healthy controls (HC) in recognizing facial negative emotions (e.g., anger, fear, sadness; Ciccarelli et al., 2022) and dynamic facial expressions, a defect contributing to worsen patients’ quality of life (Garrido-Vásquez et al., 2016).

More recently several studies addressed processing of emotional prosody in PD. The term *prosody* refers to the characteristics of spoken language such as pitch, volume, intensity, and frequency, which convey linguistic and paralinguistic information. Monrad-Krohn (1947) originally defined prosody as the component of language that carries different shades of meaning through variations in accent and tone, independently from the semantic and grammatical content. Many authors (see Liebenthal et al., 2016; Schirmer & Kotz, 2006) differentiate between linguistic functions of prosody, which allow, for example, to distinguish statements and questions, and non-linguistic functions, which allow to understand the emotion and the attitude of the interlocutor.

Emotional prosody represents a central element in social communication as it conveys information about affective states in interacting with others (Lin et al., 2018). Such a complex function relies on a set of temporo-frontal and subcortical structures, including superior temporal gyrus/sulcus (STG; STS), inferior frontal gyrus (IFG), basal ganglia, and limbic regions. Schirmer and Kotz (2006) proposed a three-stage working model for prosody comprehension in healthy individuals and outlined its cortical and sub-cortical correlates. In the sensory processing stage, bilateral auditory cortical areas analyse the acoustic features of the sound. Next, in the integration stage, auditory information is combined with emotionally relevant acoustic information in the right hemisphere, specifically in the STG and the anterior STS. Finally, in the cognition stage, the emotional information processed by the STS is made available for more complex cognitive processes, such as explicit evaluations of emotional prosody, likely involving the right IFG and the orbitofrontal cortex (OFC), or integration of emotional prosody into language comprehension, likely based on activation of the IFG in the left hemisphere. Thus, frontal and pre-frontal cortex (PFC) would be involved in processing emotional prosodic information in healthy individuals. As in PD the dopamine depletion in the nigrostriatal pathway leads to dysfunctions of subcortical-prefrontal projections (Ramesh & Arachchige, 2023), it is plausible that emotional prosody is affected in this clinical condition. More recent network models have further expanded this view, suggesting that prosody processing emerges from the dynamic interaction of distributed neural systems. For instance, Grandjean (2021) proposed a large-scale model comprising five partially overlapping neural systems: i) the thalamus and amygdala representing subcortical relevance detectors, ii) temporal areas for auditory voice construction, iii) inferior frontal regions for categorization and top-down control, iv) OFC for contextual integration, and v) cortico-subcortical loops including basal ganglia and cerebellum for temporal sequencing and action tendencies.

Several interpretations of emotional processing deficits in PD have been put forward. On one hand, there is some evidence supporting a genuine alteration of emotional processing in PD that would affect both comprehension and production. This idea is supported by data showing a dysfunction of the amygdala (Péron et al., 2013), and a linear correlation of impaired emotion recognition with loss in dopaminergic neuronal populations in PD (Lawrence et al., 2007). On the other hand, several studies maintain that the deficit in expression of emotions both through face mimic and voice could be explained by facial amimia (i.e., they suggest that rigidity, akinesia, and increased latency of facial muscle contraction could impair production of facial expressions; Scott et al., 1984), whereas dysprosody should be ascribed to a pure motor disorder of verbal articulatory production (Simons et al., 2004). According to another line of research, in patients with PD identification of linguistic prosody is likely impaired due to alterations of the subthalamic nucleus (STN). Stimulation of the STN might introduce “noise” into the auditory processing system, altering patients’ ability to use prosodic cues correctly (Kastamoniti et al., 2017). Consequently, information about the neural correlates of emotional prosody processing in PD remain inconsistent.

The present systematic review aimed to synthesise the literature investigating the neural correlates of emotional prosody processing in patients with PD using diverse techniques adopted in cognitive neurosciences. In this paper, we searched for studies employing neuroimaging, electrophysiological and brain stimulation techniques (Hallett et al., 2021; Latreille et al., 2016; Neige et al., Neige, et al., 2018) and integrated them with evidence offered by studies on deep brain stimulation (DBS) of the STN, which is an option for treating PD not responding to pharmacological therapy (Parsons et al., 2006). By analysing evidence gathered by these complementary methods, we aimed at providing a comprehensive framework putting together correlational and causative approaches. To understand the neural correlates of comprehension and production of emotional prosody, might prove useful for implementing specific treatments in patients with PD, as these deficits strongly impact on patients’ quality of life (Leite Silva et al., 2023), but also as a source of information for treating other neurological and psychiatric populations (Amlerova et al., 2022; Geraudie et al., 2023; Lin et al., 2018).

## Methods

### Search strategy

A systematic literature review was conducted following the PRISMA guidelines (Page et al., 2021).

A primary search on PubMed, Scopus, and PsycINFO databases from 1979 until August 2025 was performed. For PubMed, the following search string was used: ((“emotion*”[Title/Abstract] OR “emotion*”[MeSH Terms]) AND (“prosod*”[Title/Abstract] OR (“prosod*”[MeSH Terms] OR “voice”[Title/Abstract] OR (“voice”[MeSH Terms] OR “spoken”[Title/Abstract] OR “spoken”[MeSH Terms] OR “vocal”[Title/Abstract] OR “vocal”[MeSH Terms] OR “speech”[Title/Abstract] OR “speech”[MeSH Terms] OR “emotional prosody”[Title/Abstract] OR “emotional prosody”[MeSH Terms]) AND (“Parkinson Disease”[Title/Abstract] OR “Parkinson disease”[MeSH Terms] OR “Parkinson’s disease”[Title/Abstract] OR “Parkinson’s disease”[MeSH Terms] OR “Parkinson”[Title/Abstract] OR “Parkinson”[MeSH Terms])). This syntax searched the keywords in the title, abstract, and included the MeSH terms to ensure that relevant studies were retrieved even when keywords were not explicitly mentioned in the title or abstract. For Scopus we used the string TITLE-ABS-KEY (“emotion*”) AND TITLE-ABS-KEY (“prosod*” OR “voice” OR “spoken” OR “vocal” OR “speech” OR “emotional prosody”) AND TITLE-ABS-KEY (“Parkinson disease” OR “Parkinson’s disease” OR “Parkinson”). Finally we used the string ((title(emotion or emotional or emotions) OR abstract(emotion or emotional or emotions) OR keywords(emotion or emotional or emotions)) AND (title(prosody or prosodic or voice or spoken or vocal or speech or emotional prosody) OR abstract(prosody or prosodic or voice or spoken or vocal or speech or emotional prosody) OR keywords(prosody or prosodic or voice or spoken or vocal or speech or emotional prosody) AND title(Parkinson disease or Parkinson’s disease or Parkinson) OR abstract(Parkinson disease or Parkinson’s disease or Parkinson) OR keywords(Parkinson disease or Parkinson’s disease or Parkinson))) on PsycINFO.

### Inclusion criteria

We included in our review the studies meeting the following criteria: i) being published from 1979 to 2025; ii) being full-length articles published in English in peer-reviewed journals; iii) involving human participants only; iv) analysing the neural correlates of emotional prosody in patients with PD, by means of at least one technique of brain activity recording and/or stimulation (transcranial Direct Current Stimulation (tDCS), Transcranial Magnetic Stimulation (TMS), DBS, Electroencephalography (EEG), Event Related Potential (ERP), functional Magnetic Resonance Imaging (fMRI), Magnetic Resonance Imaging (MRI), Functional Near Infrared Spectroscopy (fNIRS); Local Field Potentials signals (LFPs), Microelectrode recording (MER) and Positron Emission Tomography (PET); v) including an emotional prosody recognition and/or production task; vi) including patients with a diagnosis of PD compared with a control group consisting of healthy individuals and/or a control patient group. We planned to exclude: i) book chapters and theses; ii) reviews and meta-analyses; iii) studies including animals; iv) studies focusing exclusively on prosody without addressing the emotional dimension; v) studies investigating the neural correlates of emotional prosody processing in PD that did not involve neural recording/imaging or stimulation techniques.

### Data extraction

From articles selected for review, we extracted the following data: authors, inclusion and exclusion criteria, sample characteristics, study design, protocol (stimulation protocol and/or recording sessions description), type of emotional prosody task, demographic information (gender, age and education), disease duration, disease stage (Hoehn & Yahr), medication status (in terms of levodopa-equivalent daily dose, LEDD; ON/OFF condition), Unified Parkinson’s Disease Rating Scale III (UPDRS III) scores and main results.

### Study selection

**Fig. 1:**
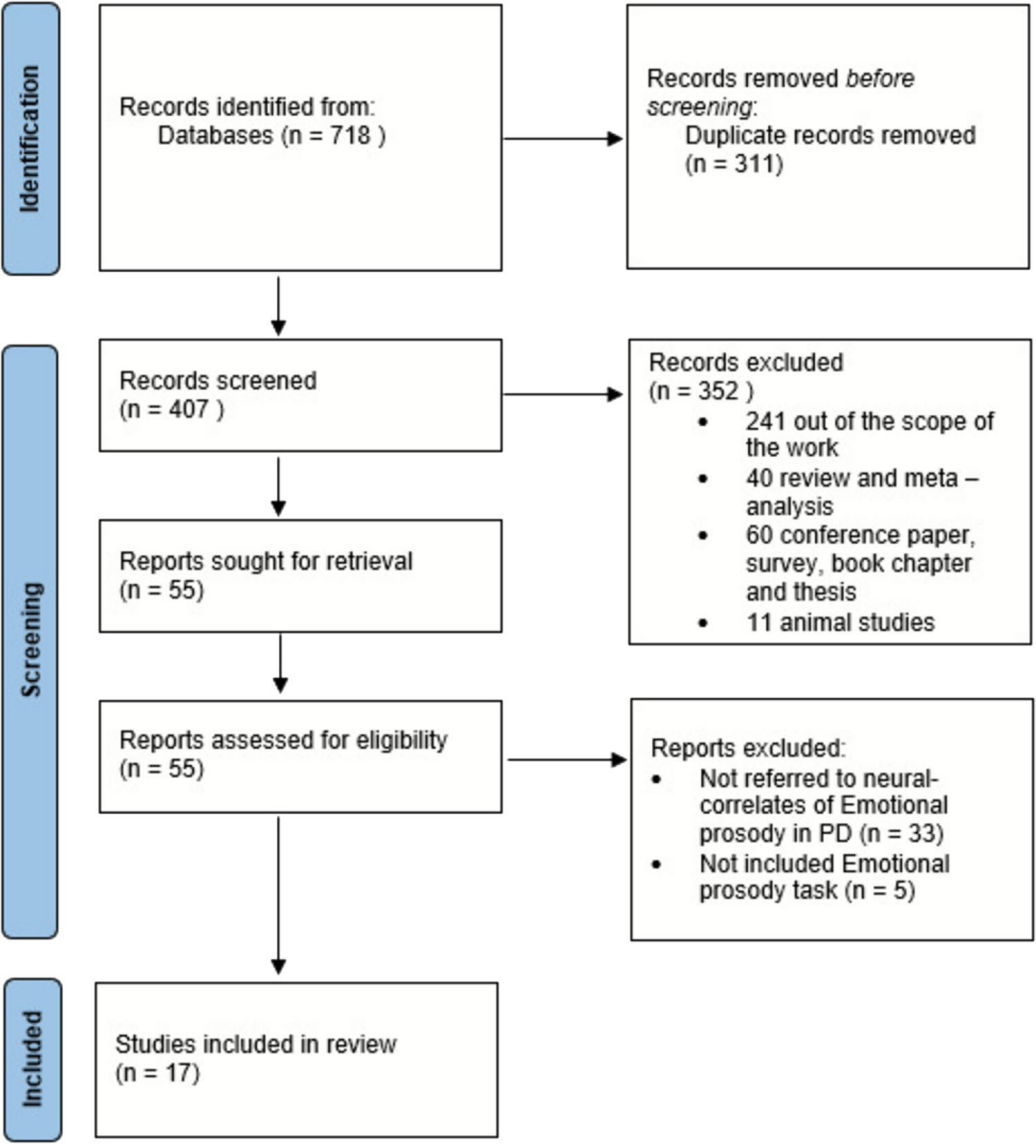
PRISMA flow diagram of selected studies addressing the neural correlated of emotional prosody in Parkinson’s disease

The primary search retrieved 718 articles. After removal of duplicate papers, the screening procedure allowed to exclude articles which were out of the scope, reviews or meta-analyses on PD not assessing emotional prosody, conference papers, surveys, book chapters, or theses; other papers were excluded because they included animals, did not address neuro-correlates of emotional prosody in PD or did not include an emotional prosody task (see Fig. 1). Seventeen studies finally entered the review.

### Quality assessment

To assess quality of the selected studies, three independent judges (SML; LC; RDB) used a modified version of the Newcastle–Ottawa Scale (NOS; Wells et al., 2011; see supplementary materials), considering appropriateness of the recruitment strategy, representativeness of the sample, outcomes, and statistical analyses. The total score ranged 0 (*unsatisfactory*) to 10 (*very good*) points. To ensure transparency, the extended NOS form used in the present review, including detailed criteria for each item, is provided in the Supplementary Materials.

## Results

### Quality evaluation

In the selected studies quality of evidence ranged from “Good” to “Unsatisfactory” (Table 1). One study was judged as “Good” (Albuquerque et al., 2014) because it used a validated emotional prosody task, described the methods accurately, used appropriate statistical tests, and clearly described the measurement of the association (including confidence intervals and/or the probability level). Twelve studies were judged as “Satisfactory” (Aiello et al., 2014; Anzuino et al., 2023; Arnold et al., 2014; Benis et al., 2020; Di Tella et al., 2021; Garrido-Vásquez et al., 2013; Jin et al., 2017; Péron et al., 2010, 2015; Schröder et al., 2006; Stirnimann et al., 2018; Voruz et al., 2020) because the methods and the tasks used were adequately explained, but they did not use validated emotional prosody tasks or described the statistical analyses incompletely. Four studies were judged as “Unsatisfactory” (Brück et al., 2011; Eitan et al., 2013; Kastamoniti et al., 2017; Péron et al., 2017) due to possible biases in analyses, limited description of methods, use of unvalidated emotional prosody tasks, poor description of the assessment of the outcome or lack of control of confounding factors.

**Table 1:** Assessment of the quality of included items according to the NOS scale

Paper	Selection					Comparability	Outcome		Evaluation
	Representativeness of the sample	Selection of the control group	Sample size	Investigation of neural correlates of emotional prosody	Measurement of emotional prosody	Confounding factors controlled	Assessment of the outcome	Statistical test	
Aiello et al., 2014	-	*	-	*	-	*	-	**	Satisfactory
Albuquerque et al., 2013	-	*	-	*	**	-	*	**	Good Study
Anzuino et al., 2023	-	*	*	*	-	-	*	**	Satisfactory
Arnold et al., 2014	-	-	-	*	*	*	*	**	Satisfactory
Benis et al., 2020	-	-	*	*	*	-	-	**	Satisfactory
Brück et al., 2011	-	*	-	*	*	-	-	*	Unsatisfactory
Di Tella et al., 2021	-	*	-	*	*	*	*	*	Satisfactory
Eitan et al., 2013	-	*	-	*	**	-	-	-	Unsatisfactory
Garrido-Vasquez et al., 2013	-	*	-	*	*	*	*	*	Satisfactory
Jin et al., 2018	-	*	-	*	*	*	*	*	Satisfactory
Kastamoniti et al., 2017	-	*	-	*	*	-	-	*	Unsatisfactory
Peron et al. 2010	-	*	-	*	*	*	*	*	Satisfactory
Peron et al. 2015	-	*	-	*	*	*	-	*	Satisfactory
Peron et al. 2017	-	-	-	*	*	-	*	*	Unsatisfactory
Schroder et al., 2006	-	*	-	*	*	-	*	*	Satisfactory
Stirnimann et al. 2018	-	-	-	*	*	*	*	*	Satisfactory
Voruz et al 2020	-	-	-	*	*	*	*	*	Satisfactory

According to *Newcastle–Ottawa Scale* (Wells et al. 2011) meaning of the total score is intended as follows: 9–10: Very Good Studies; 7–8: Good Studies; 5–6: Satisfactory Studies; 0–4: Unsatisfactory Studies

### Study characteristics

A total of 17 studies met the inclusion criteria and were classified according to methodology (DBS/LFP, EEG/ERP, fMRI/PET, MRI morphometry, and behavioural protocols) and task type (recognition vs. production of emotional prosody). Table 2 summarizes the main characteristics of each study including study type, group condition, protocol, type of emotional prosody task and main results. Table 3 includes sample size, demographic characteristics, disease duration, disease stage (Hoehn & Yahr), LEDD and UPDRS III scores, when reported.

**Table 2:** DBS and recording studies on neural correlates of emotional prosody in PD patients

Authors	Study Type	Groups/Condition	Technique used (stimulation and/or recording)	Emotional Prosody Task	Main Findings
*Studies using Deep Brain Stimulation (DBS)*					
Aiello et al., 2014	Mixed Study	PD patients were tested in 4 different conditions: (i) On Med before surgery, (ii) Off Med before surgery, (iii) On Med with stimulator never switched on 5 days after surgery and (iv) On Med/On stimulation 6 months after surgery	DBS on the STN	Auditory emotion recognition task with sentences expressed through six alternatives emotion happiness, sadness, fear, surprise, disgust, and anger	No impairment in emotional prosody recognition both before and after surgery
Albuquerque et al., 2013	Prospective longitudinal observational within study	Same group of patients before surgery and 1 year after	DBS on the STN	CATS evaluates visual recognition of 6 basic emotions (happiness, sadness, anger, fear, surprise, disgust; the task included neutral stimuli) from facial expressions and recognition of 4 emotions (happiness, sadness, anger and fear) from voice	There were no significant changes in recognition of emotional prosody, and naming of emotional prosody after DBS of the STN
Benis et al., 2020	Between study; Single session	8 LOPD patients and 6 ROPD patients	Following DBS electrode implantation in the STN, patients STN LFPs were recorded	Emotional production task in which vocal stimuli expressing anger, happiness or neutral prosody	Theta, alpha and gamma band responses to emotion were mostly bilateral with a differential pattern of response according to patient’s sides-of onset. beta-band emotional responses were mostly lateralized in the left STN for both patient groups. STN theta, alpha and gamma band responses to happiness were either absent (theta band) or reduced (alpha and gamma band) in the most affected STN hemisphere (contralateral to the side-of onset), while a late low-beta band left STN happiness-specific response was present in ROPD patients and did not occur in LOPD patients
Brück et al., 2011	Mixed study with task order balanced between subject in a Single session	PD DBS ON and PD DBS OFF (Same group of patients) and HC group (Twice time)	DBS on the STN	Auditory emotion recognition task with a happy, an angry or a neutral tone of voice in which adjectives or nouns were presented in congruent trials (same emotional meaning both in word content and prosody) or incongruent trials (meaning conveyed by prosody and word content did not match). Participants were asked to complete three tasks: i) vowel identification, in which they were asked to indicate whether a spoken word contained the letter “a” or the letter “o” or whether neither “a” nor “o” were present; ii) prosody identification, in which they were asked to judge the emotion conveyed by speakers’ tone of voice using a forced choice selection among three categories (happy, neutral or angry); iii) semantic comprehension, in which they had to classify each stimulus according to its emotional content using one of three categories (positive, neutral or negative meaning).	The processing of emotional speech is modulated by STN-DBS. Results indicated a significant ON-OFF difference for high conflict trials, explained by faster reactions observed during STN-DBS ON as compared to STN-DBS OFF
Eitan et al., 2013	Between subject study; Single session	PD group; 3 HC groups	DBS was performed on STN. MER data was acquired during all the task	MAV database which included 50 nonverbal affect bursts corresponding to 4 emotions (happiness, anger, fear, sadness, vs. neutral) recorded by 10 actors (5 males and 5 females)	Emotive auditory stimulation evoked activity in the ventral non-oscillatory region of the right STN. These responses were not observed in the left ventral STN or in the dorsal regions of either the right or left STN
Jin et al., 2018	Between study; 3 sessions (Emotional Prosody recognition)	16 PD pre-DBS group; 16 PD post-DBS group; 16 HC	DBS on the STN	1 study: MAV database (see Eitan et al., 2013) 2 study: Similar to the MAV database used in experiment 1, all participants were asked to nonverbally express five basic emotions including happiness, anger, fear, sadness, and neutral 3 study: Both subjective and objective methods were used to determine whether the materials recorded in experiment 2 conveyed the intended emotions.	The PD groups scored lower than the HC group in recognizing and expressing emotional prosody. DBS on the STN had no significant effect on the recognition of emotional prosody but had a significant effect on fear prosody expression. Pearson’s correlation analysis revealed significant correlations between performance on emotional prosody recognition tests and performance on emotional prosody expression tests in both the pre-DBS PD and post-DBS PD groups
Kastamoniti et al., 2017	Mixed randomize single-blind study; Single session in both studies	1 study: PD STN-DBS-on state; PD STN-DBS-off state. 2 study: PD STN-DBS-on state; PD STN-DBS-off; HC group	DBS on the STN	1 study: Auditory emotion recognition task with a different prosody (neutral, interrogative, sadness, sentence: neutral, interrogative, anger, admiration). 2 study: participants were asked to hear the prerecorded word/sentence and then to repeat it in exactly the same prosody (anger, admiration, interrogative, sadness and neutral)	DBS-on state negatively influences the expression of emotional prosody in the speech of PD patients
Peron et al. 2010	Between subject study Single Session	Pre-operative DBS group; Post-operative DBS group; Healthy control group	DBS on the STN	Auditory emotion recognition task was played in 4 different emotions: anger, fear, happiness and sadness, compared with a neutral condition with the visual analogic scale (VAS) to what extent each audio recording corresponded to each of the intonations	Both the pre- and post-operative groups are less accurate in recognizing emotional prosody compared to the HC. The performance of the post-operative group compared with the other two groups showed an emotional bias whereby they perceived emotions more strongly.
Peron et al. 2015	Between subject study; Single session	Pre-operative DBS group; Post-operative DBS group; Healthy control group	DBS on the STN	Auditory emotion recognition task with five different categories of prosody, five emotional (anger, fear, happiness, surprise and sadness) and one neutral, were used in the study, with the visual analogic scale (VAS) to what extent each audio recording corresponded to each of the intonations	The post-operative biased ratings on the fear scale when patients listened to happy stimuli were correlated with loudness, while the biased ratings on the sadness scale when they listened to happiness were correlated with F0. Furthermore, disturbed ratings on the Happiness scale when the post-operative patients listened to sadness were found to be correlated with F0. These results suggest that inadequate use of acoustic features following STN stimulation has a significant impact on emotional prosody recognition in PD patients, affecting the extraction and integration of acoustic cues during emotion perception
Voruz et al 2020	Between study in pre e post operative conditions	13 LPD patients; 16 RPD patients; 29 HC	DBS on the STN. All patients underwent FDG-PET scans in a resting state with their eyes open. They underwent two scans while on their antiparkinsonian medication: the first was performed 3 months before surgery, and the second 3 months after surgery, with the stimulator switched on	Auditory emotion recognition task with a set of vocal stimuli (pseudowords) in four categories of emotional prosody (anger, fear, happiness, and sadness), together with a neutral condition	LPD patients exhibited a deficit in vocal emotion recognition for neutral, anger, happiness and sadness in the preoperative condition that was normalized postoperatively. RPD patients comparably to HC in the pre-operative condition, but differed significantly on fear post-operatively
Peron et al. 2017	Single condition study	One PD group	STN LFPs and deep brain activity was recorded bipolarly from two adjacent contacts of each DBS electrode amplified, and sampled at a common rate of 1000 Hz. Signals were monitored online during the task	Auditory emotion recognition task used an emotional version of the “N back” task in response to angry, happy, and neutral prosodies, as well as to non-human synthesized stimuli	Modulation of the right STN in response to anger and happiness, as opposed to neutral prosody
*Studies using electrophysiological recording (EEG and ERP)*					
Garrido-Vasquez et al., 2013	Between subject study; 3 sessions	LPD group; RPD group; HC group	EEG was recorded (2 and 3 session), for all the task.	Auditory emotion recognition task with auditorily pseudowords corresponded to one of four emotional intonations: angry, disgusted, fearful or happy or a neutral intonation	LPD patients showed enhanced P200 amplitudes, and specific deficits were observed for disgust prosody, explicit anger processing and implicit processing of happy prosody. Lexical speech was predominantly affected while the processing of pseudo-speech was largely intact. P200 amplitude in patients correlated significantly with left motor scores and asymmetry indices
Schroder et al., 2006	Single-blind, mixed study Single Session	Oddball paradigm in passive and active conditions	ERP were recorded during all the task	Oddball recognition task in passive and active condition, with happy and sad stimuli compared with neutral stimuli	Poorer performance of PD patients in classifying emotional prosody. ERP analysis, indicating an impairment of early pre-attentive processing of emotional prosody in PD
*Studies using neuroimaging techniques (MRI, fMRI, PET)*					
Arnold et al., 2014	Mixed Study; Single se ssion in which condition	Whitin condition – same PD patient group: ON dopaminergic Med, OFF dopaminergic Med Between condition: PD group; HC group	fMRI during the sentence reading task	Emotional production task in which participants had to read semantically neutral sentences presented for 3s on a computer screen and before presentation of each stimulus, an auditory instruction indicated the intonation the participants had to use in producing the sentence (covert, overt, neutral, or happy)	Reduction of striato-prefrontal connectivity in early PD patients associated with subcortical (OFF state) or cortical (ON state) compensatory networks. During generation of affective prosody, a reduced functional coupling between the ventral and dorsal striatum was observed
Anzuino et al., 2023	Between study; Single session	15 PD patients; 15 HC	All participants were submitted to a single MRI examination in order to perform whole brain statistical analyses on grey matter volume, during the task	Emotional production task in which participants had to read short stories that were used to induce emotional states (fear, anger, disgust, sadness, and happiness, plus a neutral condition) and then had to pronounce aloud the same standard sentence according to the induced emotion	PD patients showed lower F0SD values than HC in the expression of anger. In the PD group, a positive correlation was observed between F0SD values of anger and volumes of the bilateral supramarginal gyrus, left thalamus, right inferior frontal gyrus, and amygdala. Neuroimaging results showed brain atrophy in PD patients in a widespread bilateral network, including frontal areas, left cingulate cortex, parietal areas as well as occipital cortices
Di Tella et al., 2021	Between study; Single session	15 PD patients; 15 HC	Structural MRI examination to obtain cortical and subcortical measurements	Auditory emotion recognition task with sentences in six different overtones: anger, happiness, fear, sadness, disgust, and neutral	Decoding sadness conveyed by voice was impaired in PD compared to the HC and was positively related to the volume of the dorsal striatum bilaterally and the dorsal striatum is involved in the decoding of vocal negative emotions in PD.
Stirnimann et al. 2018	Between Study	PET data :LPD group; RPD group Behavioural data: LPD group; RPD group; HC group	FDG-PET scan in a resting state in the on-dopa state.	Banse and Scherer, (1996) database included vocal stimuli consisting of meaningless speech (pseudowords) featuring 5 categories of emotional prosody (anger, fear, happiness, surprise and sadness, vs neutral) produced by male and female actors	Vocal emotion recognition was significantly poorer among patients with left-sided motor symptoms than among both right-sided patients and controls. There was no significant difference between right-sided patients and controls. At the metabolic level, positive correlations were found between the happiness recognition sub score and the metabolism of the right orbitofrontal cortex in patients with left-sided motor symptoms. A right orbitofrontal-basal ganglia coupling seems to be specifically involved in the vocal emotion recognition deficit observed in PD

*CATS* Comprehensive Affect Testing System; *DBS* Deep brain stimulation; *EEG* Electroencephalogram; *EOG* Electrooculogram; *ERP* Event-related brain potential; *F0* fundamental frequency; *F0SD* fundamental frequency standard deviation; *FDG-PET* fludeoxyglucose-18 positron emission tomography; *fMRI* functional Magnetic Resonance Imaging; *HC* Healthy controls; *LFPs* Local Field Potential Signals; *LOPD* Left- lateralized motor-onset; *LPD* Left-side Parkinson disease; *MAV* The Montreal affective voices; *Med* Medication; *MER* Microelectrode Recording; *MRI* Magnetic Resonance Imaging; *PD* Parkinson’s disease; *ROPD* Right-lateralized motor-onset; *RPD* Right-side Parkinson disease; *STN* Subthalamic nucleus

**Table 3:** Demographic, medical and symptomatic variables of PD patients and HC

Authors	Sample size	Gender M/F	Age	Education (Years)	Disease duration (Years)	Disease stage (Hoehn & Yahr)	LEDD	UPDRS III
Aiello et al., 2014	12 PD patients; 13 HC	8/4 7/6	61.2 ± 7.3 58.7 ± 7.5	11 ± 4.7 10.8 ± 3.4	10.9 ± 4	NR	NR	34.7 ± 15.6 OFF DOPA 15.2 ± 11.1 ON DOPA
Albuquerque et al., 2013	30 PD patients	18/12	62.7 ± 7.7	6.7 ± 4.6	15.85 ± 7.02	2.21 ± 0.25 (Pre-op on Medication) 2.07 ± 0.17 (Post-op on stimulation and on medication)	1148 ± 433.5 (Pre-op on Medication) 425 ± 209 (Post-op on stimulation and on medication)	16.09 ± 6.52 (Pre-op on Medication) 14.95 ± 8.68 (Post-op on stimulation and on medication)
Benis et al., 2020	14 PD patients	6/8	53.6 ± 6.7	10.79 ± 3.4	8.21 ± 2.8	NR	956.4 ± 659.4	39.46 ± 10.52 (OFF DOPA) 14.92 ± 8.39 (ON DOPA)
Brück et al., 2011	13 PD patients (STN-DBS); 11 HC	10/3 8/3	60.46 ± 8.9 60.09 ± 5.2	13.73 ± 2.67 14.41 ± 3.43	15 ± 6	NR	NR	23.15 ± 8.91 (STN – DBS ON) 56.77 ± 11.20 (STN – DBS OFF)
Eitan et al., 2013	14 PD patients; 3 HC groups (12 age and gender matched; 14 60 years old or older; 15 younger than 60 years old)	13/4	32.1 ± 7.3	11.8 ± 7.6	NR	NR	NR	79.9 ± 15.2 (OFF)
Jin et al., 2018	16 PD Pre - DBS; 16 PD Post- DBS: 16 HC	11/5 11/5 11/5	61.63 ± 8.3 61.93 ± 5.9 61.75 ± 7.5	8.94 ± 3.23 8.63 ± 2.58 8.75 ± 3.19	8.06 ± 2.29 7.56 ± 1.90 -	2.5 ± 0.73 2.4 ± 0.51 -	391.8 ± 193.1 380.5 ± 200.4	29.56 ± 15.15 28.75 ± 13.79
Kastamoniti et al., 2017	1 study: 16 PD patients; 2 study 16 PD patients; 50 HC	12/4 10/4 NR	65.1 ± 7.8 NR	NR	NR	NR	NR	NR
Peron et al. 2010	21 PD patients (pre-operative DBS group); 21 PD patients (post-operative DBS group); 21 HC	10/11 10/11 10/11	59.5 ± 7.9 58.8 ± 7.4 58.2 ± 8.0	NR	11.0 ± 3.6 11.3 ± 4.1 -	1.3 ± 0.6 (ON) 1.3 ± 1.0 (OFF)	973.6 ± 562.3 828.3 ± 523.8	9.5 ± 6.9 (ON) – 27.6 ± 13.5 (OFF) 13.7 ± 8.8 (ON) – 34.3 ± 8.0 (OFF)
Peron et al. 2015	21 PD pre-surgery; 21 PD post-surgery; 21 HC	10/11 10/11 10/11	59.5 ± 7.9 58.8 ± 7.4 58.2 ± 8.0	NR	11.0 ± 3.6 11.3 ± 4.1 -	1.3 ± 0.6 (ON) 1.3 ± 1.0 (OFF)	973.6 ± 562.3 828.3 ± 523.8	9.5 ± 6.9 (ON) – 27.6 ± 13.5 (OFF) 13.7 ± 8.8 (ON) – 34.3 ± 8.0 (OFF)
Voruz et al 2020	29 PD patients; 29 HC	16/13 16/13	56.5 ± 7.98 54.35 ± 8.5	11.30 ± 3.6 12.4 ± 2.5	11.20 ± 4.20	NR	1280.8 ± 588.5 (Pre STN DBS) 717.21 ± 562.5 (Post STN DBS)	NR
Peron et al. 2017	15 PD patients (2 removed)	8/5	53.8 ± 7.7	11.5 ± 3.1	8.0 ± 2.9	0.9 ± 1.1 (ON DOPA) 2.3 ± 0.9 (OFF DOPA)	1047.1 ± 692.0	15.7 ± 10.2 (ON DOPA) 37.0 ± 10.3 (OFF DOPA)
Garrido-Vasquez et al., 2013	22 PD patients (10 LPD and 12 RPD); 22 HC	11/11 11/11	LPD 63.9 ± 10.29 RPD 68.58 ± 6.33 HC 65.9 ± 8.2	4.5 (median) 76	6 ± 4.6 5.54 ± 3.64	2 (median) 2.25	NR	15.7 ± 4.5 13.67 ± 4.84
Schroder et al., 2006	14 PD patients, and 14 HC	7/7 7/7	62.0 ± 5.9 62.1 ± 6.6	12.7 ± 2.3 12.7 ± 1.9	4.8 ± 5.5	1.2 ± 0.4	459 ± 290	16.2 ± 6.4
Arnold et al., 2014	20 PD patients at the early stages of disease; 20 HC	12/8 12/8	63.9 ± 1.5 64.25 ± 5.5	NR	5.8 ± 0.8	1.65 ± 0.49	449.9 ± 71.0	17.4 ± 5.6 (ON) 26.05 ± 7.77 (OFF)
Anzuino et al., 2023	15 PD patients; 15 HC	9/6 6/9	69.93 ± 7.1 68.13 ± 8.3	12.2 ± 3.99 13.2 ± 3.69	41.80 ± 21.37 (months)	1.79 ± 0.38	174.31 ± 159.18	27.8 ± 10.6
Di Tella et al., 2021	15 PD patients; 15 HC	9/6 6/9	69.93 ± 7.1 68.13 ± 8.3	12.2 ± 3.99 13.2 ± 3.69	41.80 ± 21.37 (months)	1.79 ± 0.38	174.31 ± 159.18	27.8 ± 10.6
Stirnimann et al. 2018	38 PD patients; 45 HC	24/14 20/25	LPD 56.95 ± 8.97 RPD 56.74 ± 7.34 HC 54.62 ± 9.43	10.37 ± 2.99 10.21 ± 3.66 13.68 ± 3.03	12.85 ± 6.13 11.79 ± 4.19	1.27 ±0.72 (ON) – 2.32 ± 0.91 (OFF); 1.03 ± 0.97 (ON) – 2.5 ± 1.05 (OFF)	1233 ± 500.9 1314.69 ± 547.7	9 ± 6.39 (ON) – 33.11 ± 14.15 (OFF) 8.43 ± 4.97 (ON) – 34.37 ± 14.87 (OFF)

*DBS* Deep brain stimulation; *HC* Healthy controls; *PD* Parkinson’s disease; *LDP* Left-side Parkinson disease; *LEDD* Levodopa Equivalent Daily Dose; *NR* Not Reported; *RP* Right-side Parkinson disease; *STN* Subthalamic nucleus; *UPDRS III* Unified Parkinson's Disease Rating Scale

Among the 17 included studies, 11 investigated the effects of stimulation of STN in patients with PD undergoing surgery for DBS implantation (Aiello et al., 2014; Albuquerque et al., 2014; Benis et al., 2020; Brück et al., 2011; Eitan et al., 2013; Jin et al., 2017; Kastamoniti et al., 2017; Péron et al., 2010, 2015, 2017; Voruz et al., 2020). Among these 11 studies, two also used LFPs (Benis et al., 2020; Péron et al., 2017), one used the MER on STN (Eitan et al., 2013), and one used PET (Voruz et al., 2020) combining brain stimulation with brain recording. Six studies investigated neural correlates of emotional prosody by fMRI during an emotional prosody task (three studies: Anzuino et al., 2023; Arnold et al., 2014; Di Tella et al., 2021), by EEG and ERP (two studies: Garrido-Vásquez et al., 2013; Schröder et al., 2006) or by PET (Stirnimann et al., 2018). No study investigated processing of emotional prosody in PD by means of non-invasive brain stimulation (NIBS).

Most studies addressed recognition of emotional prosody (Aiello et al., 2014; Albuquerque et al., 2014; Benis et al., 2020; Brück et al., 2011; Di Tella et al., 2021; Eitan et al., 2013; Garrido-Vásquez et al., 2013; Jin et al., 2017; Kastamoniti et al., 2017; Péron et al., 2010, 2015, 2017; Schröder et al., 2006; Stirnimann et al., 2018; Voruz et al., 2020), whereas four investigated prosody production (Anzuino et al., 2023; Arnold et al., 2014; Jin et al., 2017; Kastamoniti et al., 2017).

Most studies involved patients in early-to-moderate disease stages (H&Y 1–3) with mean disease duration ranging from 7 to 11 years. In general, the studies recruited more male than female participants, reflecting the known sex distribution of PD, and no study assessed sex-related differences in prosody processing. Medication status was reported in most studies, with most testing participants in the ON state; four studies explicitly compared ON and OFF conditions (Table 3). DBS studies revealed a substantial postoperative reduction in dopaminergic medication in terms of LEDD (e.g., Albuquerque et al., 2014). A recent study by Voruz et al. (2025) provided direct evidence of differential effects of dopaminergic therapy on vocal emotion recognition depending on motor symptom asymmetry. In the next sections we will describe evidence as a function of the technique and the task employed.

### Description of the emotional prosody tasks

Most studies used a *recognition* task (Table 2). Some of these used validated databases, such as the Montreal Affective Voices database (MAV; Belin et al., 2008; i.e., Eitan et al., 2013; Jin et al., 2017), the Comprehensive Affect Testing System (CATS; Froming et al., 2006; i.e., Albuquerque et al., 2014), and Banse and Scherer’s (1996) database (Stirnimann et al., 2018). Other studies used emotional prosody recognition tasks including non-validated stimuli, such as sentences (Aiello et al., 2014; Di Tella et al., 2021; Kastamoniti et al., 2017), adjectives or nouns in congruent or incongruent trials (based on the matching between the word and the voice heard; Brück et al., 2011), or meaningless speech (pseudowords; Garrido-Vasquez et al., 2013; Péron et al., 2010, 2015; Voruz et al., 2020). One study employed an oddball paradigm in passive and active conditions where a single word was presented in three tones (happy, sad, and neutral; Schröder et al., 2006), whereas two studies used an emotional version of the “*N* back” task in response to happy, angry and neutral human prosodies, and to synthesized stimuli (Benis et al., 2020; Péron et al., 2017). In all the tasks, emotional stimuli were compared with a neutral intonation.

A few studies used an emotional prosody *production* task (Table 2), in which the patients were required to: i) reproduce the same mood heard in a recorded word/sentence (Kastamoniti et al., 2017), ii) produce a sentence with an emotional intonation corresponding to specific experimental instructions presented on a computer screen (Arnold et al., 2014); or iii) pronounce a sentence aloud after reading short emotional stories (eliciting sadness, fear, anger, disgust, and happiness, plus a neutral condition; Anzuino et al., 2023). Just one study used a validated database (MAV; Jin et al., 2017) for the production task.

### Detailed description of the studies

#### Studies on patients who underwent deep brain stimulation of the subthalamic nucleus

The 11 studies included in our review assessed the possible role of STN in recognizing and/or producing emotional prosody: i) by comparing patients before and after DBS implantation (Aiello et al., 2014; Albuquerque et al., 2014; Jin et al., 2017; Péron et al., 2010, 2015; Voruz et al., 2020); ii) by contrasting patients with DBS in ON/OFF state (Brück et al., 2011; Kastamoniti et al., 2017); iii) by comparing patients who had undergone DBS implantation with HC matched for age, gender and education (Aiello et al., 2014; Brück et al., 2011; Eitan et al., 2013; Jin et al., 2017; Kastamoniti et al., 2017; Péron et al., 2010, 2015; Voruz et al., 2020); or iv) by combining DBS and recording techniques in the same experimental session (Benis et al., 2020; Eitan et al., 2013; Péron et al., 2017; Voruz et al., 2020). Some of these studies supported an involvement of the STN in emotional prosody recognition or production (Benis et al., 2020; Brück et al., 2011; Eitan et al., 2013; Kastamoniti et al., 2017; Péron et al., 2015, 2017; Voruz et al., 2020), but others did not (Aiello et al., 2014; Albuquerque et al., 2014; Jin et al., 2017; Péron et al., 2010). Below, we first present the studies analysing accuracy in emotional prosody tasks in patients with DBS, and then the studies analysing electrophysiological data obtained from recording techniques in addition to DBS.

##### Effects on behavioural measures

Aiello et al. (2014) used a 4-session protocol in which the same group of patients was tested in four conditions and compared with a matched group of HC individuals. Patients with PD performed as well as HC in the emotional recognition task, both for accuracy and for intensity ratings. Aiello et al. (2014) concluded that STN is not particularly involved in PD’s emotional prosody recognition abilities, and that any impairment in emotional prosody recognition was not related to, or affected by, DBS surgery.

Albuquerque et al. (2014) assessed a group of patients before and one year after DBS surgery and did not find significant changes in recognition and naming of emotional prosody after DBS; these data did not support the assumption of a change in processing emotions conveyed by voice after DBS.

Brück et al. (2011) assessed emotion recognition from voice in patients with PD in both DBS ON/OFF conditions and in a control group. The PD group and the HC group did not differ in both accuracy and reaction times (RT); in PD no general difference was found between the DBS ON/OFF conditions, but during DBS-on significantly shorter RT were observed for high conflict trials, as compared to DBS-OFF.

Jin et al. (2017) assessed two groups of patients and a group of HC on two recognition tasks and one production task. Pre- and post-DBS patients scored significantly lower than HC group in recognizing the neutral stimuli, but no significant difference was observed between pre- and post-DBS groups. On the production task, the pre-DBS group expressed neutral intonation and anger less effectively than the HC group, while the post-DBS group conveyed fear less effectively than both the pre-DBS and HC groups. In synthesis, DBS on STN did not interfere with recognition of emotional prosody but affected prosody expression of fear significantly.

Kastamoniti et al. (2017) compared a group of patients with PD in DBS ON/OFF conditions, with a group of HC on a prosody perception task and a prosody expression task. The prosody perception task was administered during the DBS-ON state and after in DBS-OFF state. No significant overall differences were observed between the DBS ON/OFF states in participants with PD on the perception task. However, patients in the DBS-ON state were more likely to misinterpret neutral prosody as interrogative, whereas those in the DBS-OFF state were more likely to misinterpret interrogative prosody as neutral. Furthermore, prosody expression ratings were higher in the DBS-OFF state than in the DBS-ON state.

Péron et al. (2010) compared pre- and post-DBS patients with a group of HC on an emotional recognition task. Pre-DBS patients performed less well than the HC. No difference was observed between the pre- and post-DBS groups, or between the post-DBS and HC; additional analyses did not reveal significant differences across the three groups on single emotional categories.

Later, Péron et al. (2015) compared pre-surgery patients, post-surgery patients, and HC on an emotional prosody recognition task. The three groups performed differently in response to different emotions. Post-DBS patients rated happy stimuli as more fearful and happy stimuli as sadder. These biased ratings appeared to be related with two acoustic features (i.e., loudness and fundamental frequency [F0]). The tendency to rate happy stimuli as more fearful was associated with loudness, whereas the biased ratings toward sadness were positively correlated with F0. According to the authors, these findings supported the idea that DBS may disrupt the way acoustic cues are processed and integrated when PD patients perceive emotions in speech, thus impairing recognition of emotional prosody.

##### Effects on electrophysiological measures

A few studies reported electrophysiological data on patients undergoing DBS. By using intracranial LFPs, Péron et al. (2017) observed specific changes in the activity of the right and left STN in response to the human voice. In both hemispheres the STN showed significantly greater activity when exposed to human voice stimuli compared to synthesized stimuli, but only the response of the right STN was modulated into angry and happy prosodies.

Voruz et al. (2020) compared patients with left (LPD) or right (RPD) predominant involvement, in two sessions (pre- and post-DBS surgery), with a group of HC on an emotion recognition task. LPD patients showed biases in recognition of neutral, angry, happy, and sad intonations in the pre-operative condition but not after DBS; RPD patients, instead, scored as well as HC before DBS but showed significantly differences on fear recognition after DBS.

Benis et al. (2020), in a reanalysis of the data from Péron et al. (2017) study, compared patients with DBS with LPD or RPD; no HC group was included in the study. ERP recording revealed a strong effect of group: patients with LPD showed different activity patterns in the left STN in response to angry and happy voices compared to neutral voices. In the right STN a biphasic pattern emerged in the comparisons of angry and happy voices with neutral voices. Time frequency decomposition indicated that, overall, the onset of voices enhanced characteristic oscillatory patterns: a theta band event-related synchronization (ERS), and a desynchronization in the alpha and beta bands (ERD) relative to baseline. Oscillatory activity in the lower frequency bands (theta and alpha) was strongly influenced by the side of disease onset. In the theta band, LPD patients exhibited an emotion-specific ERS in the left STN and an earlier anger-specific ERS in the right STN. Conversely, RPD patients exhibited a significant happiness-specific ERS in the left STN and the right STN, followed by a significant anger-specific ERD. In the alpha band, patients with LPD showed an early happiness-specific ERS in the left STN followed by a later emotion-specific ERS. The right STN in LPD patients displayed an anger-specific ERS followed by a later emotion-specific ERS. RPD patients, however, showed a bilateral anger-specific ERD in the alpha band. Additionally, RPD patients showed significantly greater theta power for happiness in the right STN compared to the left ST, while LPD patients had higher alpha power for happiness in the left STN compared to the right. Beta band activity (both high and low frequencies) showed emotional modulations, primarily lateralized to the left STN. Notably, late beta bands were reduced in LPD compared to RPD. Finally, gamma band responses to happy and neutral voices were predominantly lateralized in the left STN. In summary, this study suggested that emotional processing was largely lateralized in the left STN for both patient groups. However, STN responses in the hemisphere most affected by the disease (contralateral to the side of onset) were either absent (theta band for happiness) or reduced (alpha and gamma bands for happiness); moreover, RPD patients retained a late low-beta band happiness-specific response in the left STN, which was absent in LPD patients.

Eitan et al. (2013) compared patients undergoing DBS with HC on an emotional prosody recognition task. PD patients assessed while on medication (24 h before surgery) were less accurate in recognising positive and neutral intonations than the HC. MER implanted during DBS surgery revealed stronger responses to emotional stimuli in the ventromedial, non-oscillatory, region of the right STN than in its dorsolateral, oscillatory, region; no change in activity was observed either in the ventral or the dorsal region of the left STN. These findings suggested that the ventral non-oscillatory regions of the STN play a role in non-motor functions, with a notable hemispheric asymmetry as the right STN appears to be particularly associated with emotional processing, thus contributing to the emotional changes observed in PD.

##### Comment

Taken together evidence from investigation of emotional prosody processing in patients with PD undergoing DBS appears to be quite inconsistent. Indeed, although most studies would support a contribution of the STN in perception and production of emotional prosody in PD (Benis et al., 2020; Brück et al., 2011; Eitan et al., 2013; Kastamoniti et al., 2017; Péron et al., 2015, 2017; Voruz et al., 2020), some studies did not support such involvement (Aiello et al., 2014; Albuquerque et al., 2014; Jin et al., 2017; Péron et al., 2010).

#### Electroencephalography and event-related potential studies

Two studies analysed the cerebral electric activity during an emotional prosody recognition task. Schröder et al. (2006) assessed ERP correlates of emotional prosody processing in patients with PD and in a group of HC. At the behavioural level, RT did not differ between the two groups, which both showed faster responses to neutral stimuli. Instead, HC’s accuracy was at ceiling for happy and sad stimuli, whereas patients with PD showed more errors for sad intonation. At the electrophysiological level, ERP analysis showed a significantly lower P3b amplitude for happy stimuli in PD than in HC, with no significant between-group differences for sad stimuli. P3b, a subcomponent of the P300, is a positive wave peaking at around 300 ms, elicited by improbable events: the less probable the event, the larger the P3b (Polich, 2008). Additionally, the HC group showed a lower P3b amplitude for sad stimuli compared with happy. This pattern of findings would suggest an impairment of early pre-attentive processing of emotional prosody in PD.

Garrido-Vásquez et al. (2013) examined emotional prosody recognition during EEG recording in patients with LPD or RPD and in a group of HC. LPD patients performed significantly worse than HC, whereas the other comparisons between groups were not significant. EEG analysis revealed that LPD patients showed that amplitude of P200 (a positive potential peaking around 200 ms after a stimulus) was larger than in the other groups. During explicit processing of lexical anger, LPD patients exhibited a larger P200 amplitude at midline and right-central electrodes compared to HC and RPD. During implicit processing of lexical disgust, LPD patients showed larger P200 amplitude in two posterior regions compared to HC and RPD. Similarly, when processing disgust expressions in pseudospeech, LPD patients had larger P200 amplitudes in the right posterior region than the other groups. Last, during the explicit task involving lexical sentences with happy intonation, LPD patients showed increased P200 amplitudes at midline electrodes.

##### Comment

These findings highlighted specific deficits in LPD patients related to explicit and implicit processing of specific emotional intonations, as evidenced by the distinct P200 responses. Taken together with the results by Schröder et al. (2006), these data suggest that emotional prosody recognition deficits in PD may involve both early (P200) and later (P3b) stages of auditory-emotional processing.

#### Structural and functional imaging studies

Among the included studies, one employed fMRI during an emotional prosody production task performed within the scanner (Arnold et al., 2014) while two used structural MRI to correlate volumetric measures with performance on production (Anzuino et al., 2023) or recognition (Di Tella et al., 2021) tasks administered outside the scanner; one study correlated brain metabolism with performance on an emotional prosody recognition task (Stirnimann et al., 2018).

Arnold et al. (2014) compared a group of patients with early-stage PD (without overt speech or voice difficulties, i.e., prior to onset of typical parkinsonian speech symptom) in ON or OFF dopaminergic medication with a HC group during an emotional prosody production task. On behavioural level there were no significant differences between PD patients and HC or between PD patients ON or OFF medication when producing speech with neutral intonation. However, fMRI revealed inverse brain activation patterns during preparation and execution of overt reading with happy vs. neutral intonation. In PD patients the left dorsolateral prefrontal cortex (DLPFC) was overactivated during the preparation phase for affective prosody compared to neutral, but underactivated during the production phase compared to HC. Conversely, the left superior parietal lobule (SPL) was underactivated during preparation but overactivated during production of affective prosody. In addition, PD patients demonstrated increased activation in the left occipitotemporal junction (OTJ) during the preparation phase for emotional prosody. No differences in prosody-related brain activity were observed between the ON and OFF medication conditions. Early-stage PD was associated with reduced connectivity between the striatum and PFC, which led to compensatory activation in subcortical regions in the OFF state and cortical regions in the ON state. During the preparation phase for emotional prosody, the hypo-activated left SPL showed weaker functional connectivity with the left IFG in PD patients (both on and off medication) compared with HC. However, during prosody production, the overactivated left SPL showed stronger connectivity with the left supplementary motor area (SMA). The overactivation of the left DLPFC during preparation in PD was linked to increased striatoprefrontal connectivity, involving the left DLPFC, SMA, IFG, and caudate nucleus. Additionally, during the OFF medication state, there was reduced preparatory connectivity between the right limbic ventral striatum and the sensorimotor dorsal striatum. Finally, during the production of happy intonation (execution phase), PD patients in the OFF state displayed less efficient connectivity between the right STS and the right sensorimotor dorsal striatum compared with controls. Arnold et al. (2014) did not report robust correlations between fMRI activations and acoustic/perceptual parameters.

In Anzuino et al. (2023) PD patients and HC underwent MRI and performed an emotional prosody production task. The goal was to investigate the relationships between the standard deviation on fundamental frequency (F0SD), a measure of pitch variability in speech, and the volume of specific grey matter regions. PD patients showed reduced pitch variation, i.e., lower F0SD values, than HC when expressing anger. Neuroimaging results revealed widespread bilateral brain atrophy in PD patients, affecting frontal regions, the left cingulate cortex, parietal areas, and occipital cortices. Importantly, in the PD group lower F0SD values for anger were correlated with reduced volumes in the bilateral supramarginal gyrus, left thalamus, right IFG, and amygdala. The authors found associations between F0SD (anger) and volumes in bilateral supramarginal gyrus, left thalamus, right IFG, and right amygdala.

Di Tella et al. (2021) administered an emotional prosody recognition task to a group of patients with PD and a group of HC who underwent a structural MRI investigation. Patients with PD were impaired in decoding sadness compared to HC and this impairment was correlated with reduced volume in the left and right dorsal striatum. As in the early stages of PD dopamine depletion can lead to atrophy in the striatum (Sterling et al., 2013), these findings support the idea that the dorsal striatum plays a role in recognizing negative emotions in vocal expressions in PD. The authors reported a selective relationship between sadness recognition and bilateral dorsal striatum volumes.

Stirnimann et al. (2018) assessed recognition of emotional prosody in LPD and RPD in on-dopa state (compared to HC) and correlated performance with data obtained by resting-state Fluorodeoxyglucose PET (FDG-PET). Emotion recognition was significantly poorer in LPD than in both RPD and HC, whereas RPD performed as well as HC. On a metabolic level, happiness recognition in LPD patients was positively correlated with the metabolism of the right OFC. Additionally, the coupling between the right OFC and the basal ganglia was specifically linked to the emotion recognition deficits observed in PD.

##### Comment

Multimodal evidence converges on phase-dependent alterations in activity and connectivity during prosody production (DLPFC/SPL and prefrontal-parietal-striatal circuits), with no clear effects of the dopaminergic on/off state. Performance (pitch variability and recognition, particularly of sadness) is associated with distinct structural/metabolic markers (supramarginal gyrus, IFG, thalamus, amygdala, dorsal striatum, and right OFC), suggesting partially differentiated neural bases for production and recognition.

## Discussion

The aim of this review was to analyse the evidence on the neural correlates of emotional prosody processing in PD. Most studies included patients undergoing intervention for DBS of the STN, and a few studies used brain recording techniques. No study employed NIBS or recording through fNIRS. Overall, the results obtained by using such complementary techniques provided evidence about brain areas and networks involved in emotional prosody production and recognition in patients with PD. The evidence gathered here seems to be consistent with the view that emotional prosody deficits in PD are not simply secondary to motor impairment, as shown by converging findings from tasks with minimal motor demands and by measuring neural responses prior to overt vocal production. For instance, in the fMRI study by Di Tella et al. (2021) patients with PD were impaired in recognising sadness in a listening task where motor involvement was negligible (Péron et al., 2010). Similarly, Stirnimann et al. (2018) reported altered metabolic coupling between the right OFC and the basal ganglia during prosody recognition, independent of speech motor output. Electrophysiological studies have also demonstrated ERP alterations occurring before vocal responses, indicating that abnormal emotional prosody processing in PD emerges already at the perceptual-cognitive stage (Garrido-Vásquez et al., 2013). Thus, the present systematic review would support the interpretation that emotional prosody deficits in PD reflect primary neurocognitive disturbances of affective communication networks, rather than being mere consequences of impaired articulation or voice modulation (Dan et al., 2019; Garrido-Vásquez et al., 2016; Lawrence et al., 2007; Péron et al., 2013).

In our review, only four studies used an emotional prosody *production* task (Anzuino et al., 2023; Arnold et al., 2014; Jin et al., 2017; Kastamoniti et al., 2017). These studies highlighted that PD impacts prosody production and that some factors, such as pharmacological treatment and DBS, may influence these effects. Anzuino et al. (2023) demonstrated that patients with PD, compared to HC, show a reduction in F0SD variability, particularly evident in the expression of anger. This suggests that patients have a reduced ability to flexibly use prosody to express more intense or dynamic emotions. From another perspective, Arnold et al. (2014) reported that patients with PD, even in the early stages and without evident voice or speech symptoms, exhibit significant differences in brain activity during prosodic production. Altered brain activation during the preparation and execution of emotional prosody compared to the neutral condition, with a reversal in cortical activation patterns, suggest that there is an abnormal compensatory effort to prepare for producing affective intonations. Both studies highlighted the involvement of specific brain areas, such as the DLPFC and the supramarginal gyrus, in the regulation of emotional prosody. However, while Anzuino et al. (2023) pointed out a positive correlation between variability of prosodic frequency and the volume of these cortical regions, Arnold et al. (2014) showed that the functional activation of these areas during prosodic preparation is altered in patients with PD, suggesting that not only structural degeneration but also functional dysregulation could contribute to these difficulties. Beyond motor impairment, reduced prosodic expressivity may also reflect higher-order non-motor symptoms commonly observed in PD, such as affective flattening, apathy, and alexithymia. These conditions, which involve difficulties in emotional generation and awareness, are consistent with the observed reduction in pitch variability (F0SD) and with correlations between prosodic deficits and volumes in limbic and frontal regions (Anzuino et al., 2023).

The effect of pharmacological treatment is another key aspect. Arnold et al. (2014) found no meaningful differences in prosodic production between dopaminergic ON and OFF states, indicating that motor improvements do not automatically extend to emotional voice. By contrast, DBS appears to have more specific effects: Jin et al. (2017) and Kastamoniti et al. (2017) report that stimulation of the STN can alter the expression of certain emotions, most notably fear, and increase errors in neutral and interrogative intonation. Taken together, these findings suggest that patients with PD exhibit persistent deficits in emotional prosody arising from both structural–functional degeneration (e.g., inferior frontal gyrus, thalamus, amygdala) and compensatory, atypical cortical activations (e.g., DLPFC, SPL), as already observed by Arnold et al. (2014). Neither pharmacotherapy nor DBS fully restores prosodic abilities; in some cases, DBS may even exacerbate specific impairments of emotional expression. This underscores the complex interaction between therapeutic interventions and the cognitive-emotional mechanisms that support prosody across disease stages.

### A cortico-subcortical network in emotional prosody in PD

Investigations on prosody recognition using brain recording techniques provided a fine-grained information about the network-level mechanisms supporting emotional prosody. EEG and ERP studies clarified the temporal dynamics of processing and showed alterations in early components such as P200 and P3b (Pfurtscheller et al., 1999), pointing to deficits in sensory gating and attentional allocation with the first 200–300 ms (Garrido-Vásquez et al., 2013; Schröder et al., 2006). Notably, Schröder et al. (2006) emphasised difficulties with positive emotions (happy), while Garrido-Vásquez et al. (2013) focused on negative emotions (anger, disgust) and observed lateralised activity, particularly in LPD, suggesting hemisphere-specific deficits. fMRI and MRI studies highlighted the engagement of a distributed cortico-subcortical network. Arnold et al. (2014) and Anzuino et al. (2023) found abnormal activation and connectivity patterns in the DLPFC, SPL, OTJ, and supramarginal gyrus during emotional prosody production, suggesting compensatory recruitment of higher-order cortical regions. Di Tella et al. (2021) further emphasized the role of the dorsal striatum in decoding sadness, supporting the involvement of basal ganglia. These findings indicate that emotional prosody processing deficits in PD are linked to specific neural pathways as a function of the type of emotional prosody task and of the lateralization of motor symptoms. The PET study (Stirnimann et al., 2018) reported worse performance and reduced metabolic activity, mainly involving the right OFC, in LPD compared with both RPD and HC, which could be related to impaired coupling with basal ganglia. Importantly, neuroimaging evidence also implicated the cerebellum in emotional prosody processing. Voruz et al. (2020) demonstrated, using PET, that bilateral cerebellar activity changes pre- and post-DBS, and that these changes correlate with performance in prosody recognition. This supports the idea that the cerebellum is part of a temporo-fronto-cerebellar loop contributing to temporal prediction and affective modulation, as it has been also described by brain stimulation studies in healthy participants (Luciano et al., 2025; Panico et al., 2024). Taken together, these methodologies converge in supporting that the early temporal abnormalities detected by ERP correspond to spatially localised alterations in temporal, frontal, and subcortical nodes (including ventromedial STN oscillatory tuning to emotional stimuli; Benis et al., 2020), thus suggesting a stepwise progression from early sensory-attentional disruption to inefficient integration and regulation within fronto-temporo-basal ganglia-cerebellar networks. This picture aligns with large-scale network models (Grandjean, 2021), indicating that prosodic impairments in PD reflect disrupted cortico-subcortical connectivity, particularly within circuits linking STG/STS, basal ganglia, PFC, and cerebellum, thereby accounting for variability across tasks and emotions and for differential effects of dopaminergic therapy and DBS. Overall, PD is consistently associated with impaired emotional prosody, with patterns of activation, connectivity, and metabolism varying by emotion, task type, and motor-symptom lateralisation.

### A role of the STN in emotional prosody in PD

DBS of the STN represents a substantial therapeutic advance for severely disabled PD patients (Temel et al., 2006; Parsons et al., 2006). However, mixed results are available on its impact on emotional prosody. The incongruencies across the studies could be related to differences in the protocols used for DBS, as well as in the task or the experimental conditions being used. First, DBS ON/OFF conditions provide a dimension of comparison between the studies. Brück et al. (2011) and Kastamoniti et al. (2017) examined patients’ performance in both conditions, revealing that stimulation can have a significant, but not always predictable, impact on emotional prosody. Indeed, Kastamoniti et al.’s (2017) study, that investigated both emotional prosody and linguistic prosody, found that patients in the DBS-ON state showed specific errors in identifying linguistic prosody, especially interrogative prosody, compared to the DBS-OFF condition. These results suggest that STN stimulation might introduce a “noise” into the auditory processing system, altering patients’ ability to use prosodic cues correctly, although quality evaluation showed some methodological issues which may have affected the results. A few well-designed studies compared pre- and post-DBS patients (Aiello et al., 2014; Albuquerque et al., 2014; Jin et al., 2017; Péron et al., 2010, 2015; Voruz et al., 2020) and reported mixed results as well. Some authors (Aiello et al., 2014; Albuquerque et al., 2014; Péron et al., 2010) did not report significant changes in the ability to recognize emotional prosody post-surgery and suggested that both in pre- and post-operative conditions patients with PD are less accurate than HC in recognizing emotional prosody. This could mean that stimulation of the STN did not produce any change in patients’ performance. The remaining studies found specific biases in interpreting emotions post-DBS. Jin et al. (2017) demonstrated that PD patients in post-DBS condition showed lower scores in expressing fear prosody, whereas Péron et al. (2015) suggested that inadequate processing of acoustic features after STN stimulation could affect emotional prosody recognition. When comparing LPD and RPD, Voruz et al. (2020) found that LPD patients showed impairments in recognizing a range of vocal emotions before surgery, but these deficits recovered after surgery. Thus, evidence was inconsistent across studies, as the first two studies highlighted that following DBS patient’s performance could be impaired, while the last study supported the idea that DBS could improve accuracy in emotional prosody recognition tasks. Other studies suggested the presence of specific correlations between side and regions of STN and emotional processing. Recording techniques such as ERP (Benis et al., 2020) and MER (Eitan et al., 2013) provided fine-grained evidence on the possible neural correlates of emotional prosody. Benis et al. (2020) observed a lateralization of emotional responses in the STN, with specific modulations for emotions like anger and happiness. This observation would suggest that the STN not only participates in emotional processing but does so in a lateralized manner, with different responses between the left and right hemisphere. Similarly, Eitan et al. (2013) found that the ventral region of the right STN was particularly active during the processing of vocal emotions, indicating functional specialization within the STN. Other evidence further supported the lateralized effects of DBS on emotional prosody. Benis et al. (2020) demonstrated that emotional responses were predominantly lateralized in the left STN for both LPD and RPD, with significant power differences in theta and alpha bands for happiness between the two hemispheres. This seem to suggest a complex interaction between the emotional stimuli and the lateralized motor symptoms of PD, affecting how emotional prosody is processed, whereas Voruz et al. (2020) found that RPD patients had specific deficits in fear prosody recognition postoperatively, which was not observed in LPD patients. From these studies the impact of DBS on emotional prosody does not seem to be uniform across all emotions and might vary based on the side of symptom onset. Consistent with this view, Voruz et al. (2025) demonstrated that dopaminergic therapy can have opposite effects on emotional prosody recognition depending on the side of motor symptom onset: it improved performance in RPD patients but worsened fear and anger recognition in LPD patients, suggesting an “overdose effect” in relatively preserved dopaminergic pathways. This observation highlights the importance of considering both dopaminergic state and lateralization when evaluating emotional prosody processing in PD and designing future studies.

It is important to underline that the interpretation of available data requires an anatomically grounded account of the STN and its functional subdivisions. Evidence from intraoperative recordings and neuroanatomical studies suggests that the STN is not a homogeneous structure, but is organised into partially segregated sensorimotor, associative, and limbic territories (Benis et al., 2020; Eitan et al., 2013). The ventromedial limbic sector is strongly connected via the ventral pallidum and mediodorsal thalamus to OFC and cingulate cortex, regions critically involved in affective evaluation and social cognition (Albuquerque et al., 2014; Benis et al., 2020; Brück et al., 2011). Within this framework, DBS-induced alterations in prosody should not be interpreted solely as by-products of motor loop modulation. Rather, they may reflect the involvement of limbic circuits within the STN and their projections to prefrontal networks. Studies reporting interpretive biases or reduced sensitivity to affective prosodic cues after STN-DBS (e.g., Péron et al., 2015, 2017) are consistent with such a view, as they suggest a disruption of top-down affective integration mechanisms mediated by orbitofrontal and medial prefrontal regions. This perspective highlights the need for future DBS research to adopt a network-based approach, carefully distinguishing the impact of stimulation on sensorimotor versus limbic territories of the STN. Such an anatomically informed interpretation may help reconcile heterogeneous behavioural findings and clarify the pathways through which DBS modulates emotional prosody in PD.

In conclusion, DBS-STN in PD shows variable effects on emotional prosody, influenced by symptom lateralization, DBS ON/OFF conditions, and task characteristics. The large variety of stimuli chosen in the different studies might help explaining result variability. The studies examined here indicated that the involvement of the STN in emotional prosody processing is modulated by individual and situational variations. Importantly in the attempt to understand the heterogeneity of these findings and with the aim of orienting future work on the topic, the quality of evaluated studies should be also considered as some methodological drawbacks may have limited reliability of observed findings (Brück et al., 2011; Eitan et al., 2013; Kastamoniti et al., 2017; Péron et al., 2017).

### Contributions of frontal and temporal regions

Neuroimaging studies of emotional prosody processing in PD consistently point to the involvement of both frontal and temporal cortices, yet their functional roles are not always clearly differentiated. Evidence suggests that superior temporal regions, particularly the STG and STS, support the perceptual-affective integration of prosodic cues, enabling the extraction of emotional meaning from acoustic features. This aligns with ERP findings that point to early abnormalities in auditory-perceptual processing (Garrido-Vásquez et al., 2013; Schröder et al., 2006). In contrast, frontal regions such as the IFG, DLPFC, and medial PFC appear to subserve higher-order inferential and regulatory processes. For example, the IFG has been implicated in the integration of prosodic and linguistic information and in the generation of expressive intonation (Anzuino et al., 2023), while the DLPFC and medial PFC are more closely linked to working memory, cognitive control, and the top-down modulation of emotional responses. Alterations observed in patients with DBS (Péron et al., 2015, 2017), where interpretive biases emerge, further underscore the role of prefrontal regions in monitoring and regulating prosodic judgements. Taken together, these findings suggest a functional division of labour: temporal regions might underpin perceptual-affective decoding, while frontal regions may contribute to inferential, executive, and regulatory aspects of prosody processing. Clarifying this distinction provides a more refined understanding of the cortical architecture supporting emotional communication and may help to explain why deficits in PD manifest not only as impaired recognition, but also as difficulties in interpreting and regulating prosodic cues flexibly.

### Relationships between the present results and available frameworks on emotional prosody

As recalled above, Schirmer and Kotz’s (2006) model describes three stages for auditory processing of emotional prosody, outlining a sequence of neural processes involving both cortical and subcortical areas in healthy individuals. This model is useful for interpreting the findings of the present review, as it provides a theoretical framework in which to place the alterations observed in patients with PD. First, in Schirmer and Kotz’s (2006) model, the sensory processing stage involves the acoustic analysis of emotional prosody by bilateral auditory cortical areas. The studies included in the present review, using EEG and ERP techniques, highlighted alterations in early ERP components (P200 and P3b) in PD patients (Schröder et al., 2006), suggesting that the early stages of sensory processing of emotional prosody are impaired. According to the model, altered acoustic processing in the cortical auditory areas could be influenced by the dysfunction of cortical and subcortical circuits in PD. The second stage of the model involves the integration of acoustic information with emotional content in the STG and the STS, predominantly in the right hemisphere. The present review highlighted evidence of lateralization of emotional responses in the STN, with different effects between the two hemispheres and a sizeable variability based on the lateralization of motor symptoms in PD. This could support the view that impaired integration of emotional information may be ascribed to dysfunctional interaction between temporal cortical areas and subcortical structures, such as the STN, thereby hampering correct processing of emotional prosody. The third stage of the model involves higher-order cognitive processes, such as the explicit judgment of emotional prosody, mediated by the IFG and the OFC. The present review highlighted that patients with PD show deficits in using acoustic features and are affected by specific emotional biases post-DBS, such as reduced accuracy in recognizing emotions like fear and happiness. These deficits may be linked to dysfunctions in subcortical-prefrontal projections due to dopamine depletion in the nigrostriatal pathway, impairing the capacity for emotional judgment and integration in frontal areas, as suggested by Schirmer and Kotz’s (2006) model.

Interestingly, our synthesis converges also with more recent network-based frameworks of emotional prosody processing. In particular, Grandjean (2021) provided an updated and comprehensive framework of the brain networks supporting emotional prosody, emphasizing that prosody decoding emerges from the interplay between temporo-frontal cortical regions, basal ganglia, limbic structures, and the cerebellum. This model complements the stage-based approach of Schirmer and Kotz (2006) by highlighting large-scale connectivity and dynamic interactions across systems, rather than strictly sequential processing. Interpreting the findings from this systematic review within this perspective suggests that PD-related deficits are not confined to isolated nodes (e.g., STG or IFG), but rather reflect dysfunctional communication between auditory, limbic, and frontal regions, potentially leading to impaired integration of emotional cues and reduced compensatory plasticity.

Putting together the available evidence, the results of this review can be mapped onto distinct yet interconnected phases of emotional prosody processing, which also reflect cognitive and neuropsychological domains typically impaired in PD (Schirmer & Kotz, 2006). Overall, converging evidence supports a multistage, network-based disturbance in PD: early sensory-attentional anomalies in the *Acoustic Analysis Stage* (Stage 1) co-occur with altered integration within temporo-basal ganglia–OFC circuits in the *Emotional Integration Stage* (Stage 2) and with frontal executive/regulatory inefficiencies during explicit evaluation in the *Cognitive Evaluation Stage* (Stage 3). These findings suggested that a purely motor account is insufficient, highlighting dysfunctional communication across cortico-subcortical circuits rather than isolated regional failure.

These considerations align with research on Huntington’s disease (HD), another degenerative disease of the basal ganglia. Dogan et al. (2014) found reduced fMRI responses in HD, relative to HC, in temporal and prefrontal cortices and in subcortical regions (including amygdala, hippocampus, striatum, insula, and cingulate). Evidence on emotional prosody remains limited: Speedie et al. (1990) reported impaired comprehension of both linguistic and emotional prosody in HD. Future studies should directly compare PD and HD to analyse the neural substrates of emotional processing and prosodic deficits. Comparative approaches will be particularly important for disentangling disease-specific versus shared neural mechanisms. Recent evidence suggests that basal ganglia dysfunction impacts social and affective cognition in several movement disorders, including HD, and may differentially affect the neural systems underlying emotional prosody (Di Tella et al., 2025; Eddy, 2025). Incorporating such comparative perspectives could help clarify whether emotional prosody deficits in PD reflect disruption of domain-general socioaffective circuits, or whether they are linked to PD-specific alterations in cortico-subcortical loops. This line of research holds promise both for refining theoretical understanding of prosody processing and for identifying disorder-sensitive markers of socioemotional impairment.

## Conclusions

In conclusion, the studies reviewed here highlight the complexity of emotional prosody perception and production in PD, with differences depending on symptom lateralization, task types, and treatment modalities. STN-DBS appears to have variable effects on prosody, with some studies reporting an impairment in the ability to process or express emotions, while cortical and subcortical network alterations may also play a role in the emotional prosody abilities in PD. Overall our analysis suggests that emotional prosody deficits in PD are influenced by both sensory processing and cognitive-emotional integration, but available evidence points to the need for more targeted interventions to address these specific impairments.

Several limitations emerge from the reviewed literature, which may envisage specific directions for future studies. First, for DBS and recording studies, different experimental tasks have been adopted, assessing recognition or production of emotional prosody. This heterogeneity may contribute to the inconsistencies among the studies, thus warranting use of standardised tasks in larger samples of participants to comprehend the involvement of cortico-subcortical structures in emotional prosody processing. A standardized set of stimuli (such as the EMOVO CORPUS; Costantini et al., 2014) could reveal useful for evaluating accuracy in emotional prosody recognition tasks; for assessing patients’ responses in emotional prosody production tasks, standardized measures for phonetic and acoustic analysis (Boersma & Van Heuven, 2001), could complement subjective evaluation.

Second, a relevant issue is that no study used NIBS techniques for addressing emotional prosody processing in PD. For instance, TMS could be used to temporarily disrupt or enhance activity in areas such as the STG or IFG, which are assumed to play critical roles in the sensory, integrative, and cognitive stages of emotional prosody processing (Schirmer & Kotz, 2006). By selectively stimulating these regions, it could be possible to better understand their specific contributions and the impact of PD-related dysfunctions on emotional prosody, also elucidating the interactions between cortical and subcortical structures involved in emotional prosody, as shown in previous studies on healthy individuals (Panico et al., 2024). Crucially, combining NIBS with neuroimaging techniques could also reveal how modulating cortical regions affects subcortical structures, such as the STN and vice versa (Bergmann et al., 2016). NIBS could also contribute to clarify the differential roles of the left and right hemisphere and assess how lateralized brain dysfunctions in PD affect emotional prosody. This could be particularly useful in understanding the reason why emotional deficits related to fear or happiness recognition are more evident in patients with left or right hemisphere motor dominant PD. Moreover, it is also worth mentioning that NIBS could have therapeutic applications for improving emotional prosody deficits in PD patients. For instance, tDCS has been shown to enhance cognitive and emotional processing in various neurological conditions (Fregni et al., 2021). Beyond their value as experimental probes, NIBS techniques could also serve as adjunctive rehabilitation strategies. By enhancing the excitability of fronto-temporal networks, NIBS could be combined with behavioural interventions such as speech therapy or affective communication training to reinforce learning and promote long-term plasticity. Preliminary studies in other neurological populations (e.g., post-stroke aphasia, affective disorders) have already demonstrated that pairing NIBS with targeted training improves both recognition and production of emotional cues (Darkow et al., 2017; Shah-Basak et al., 2023), suggesting that similar synergistic approaches may be feasible in PD. Future work should therefore systematically investigate the translational potential of NIBS for probing neural mechanisms, but also for mitigating prosodic deficits in these patients. In addition to the already noted absence of studies employing NIBS, there is also a lack of works using highly ecological brain recording techniques, such as fNIRS. fNIRS could be particularly valuable because it would allow the examination of emotional prosody during face-to-face interactions or dynamic dialogic tasks (e.g., Panico et al., 2021).

Third there is a lack of data on evolution of emotional prosody deficits in PD. Longitudinal studies will help to track changes in emotional prosody over the progression of PD and provide deeper insights about how disease progression affects the neural circuits involved in emotional processing. A further limitation concerns the methodological quality of the available studies. Indeed, according to NOS-based evaluation, only a small proportion of studies reached high quality levels, suggesting that the reliability of findings may vary across methodologies and highlight the need for future research with larger and statistically justified samples, greater control of confounding factors, and more standardised outcome measures.

A last closing remark deals with the clinical and social implications of emotional prosody deficits in PD. Impairments in recognising and producing emotional prosody in PD have profound consequences for patients’ social functioning, often leading to misunderstandings in daily communication strained interpersonal relationships (Gnerre et al., 2023) and social withdrawal. These alterations may also challenge therapeutic interactions, limiting clinicians’ and caregivers’ ability to interpret patients’ affective states. For these reasons, prosody processing should be regarded not only as a research construct but also as a potential symptom informing about disease progression and a target for tailored interventions.

## Supplementary Information

Below is the link to the electronic supplementary material.

Supplementary file1 (DOCX 16 kb)

## Data Availability

Not applicable.
