# Supplementary material for: Neural correlates of emotional prosody in Parkinson’s disease: A systematic review
Source: Cogn Affect Behav Neurosci. 2026 Jan 8;26(3):798–823. doi: 10.3758/s13415-025-01379-w (PMC13260268; doi:10.3758/s13415-025-01379-w)
Supplement: Supplementary file 1 — Supplementary file1 (DOCX 16 kb) [file 13415_2025_1379_MOESM1_ESM.docx]

| Selection -  Maximum 6 stars | Representativeness of the sample:  **a)** Truly representative of the average in the target population ** (all subjects or random sampling)  **b)** Somewhat representative of the average in the target population * (non-random sampling)  **c)** Selected group of users  **d)** No description of the sampling strategy |
| --- | --- |
|  | Selection of the control group:  **a)** matched sample for age and education *****  **b)** no description of the derivation |
|  | Sample size:  **a)** Justifies and satisfactory (description of power analysis) *** b)** Not justified |
|  | Investigation of the neural correlates of emotional prosody: **a)** Well described measurement techniques ***** (EEG; ERP; fNIRS; fMRI; MRI; PET; MER; NIBS) **b)** No description or insufficient description in methods section |
|  | Measurement of emotional prosody: **a)** Validated measurement tool ****** (CATs; MAV; Prosody Assessment Rating Scale) **b)** Non-validated measurement tool, but the tool is described and adequate (comparison between emotions and neutral) *****  **c)** No description of the measurement tool |
| Comparability - Maximum 1 star | Confounding factors controlled. **a)**Data/ results adjusted for relevant predictors/risk factors/confounders e.g. age, sex, time post-injury or diagnosis, medications *** b)**Data/results not adjusted for all relevant confounders/risk factors/information not provided |
| Outcome - Maximum 3 stars | Assessment of the outcome:  **a)** Independent or blind assessment (double-blind) ****  b)** single blind or not validated but well descripted methods ***  c)** no description  Statistical test:  **a)** The statistical test used to analyse the data is clearly described and appropriate, and the measurement of the association (including confidence intervals or effect size; non-significant effect; M and SD; exact probability level, and statistical test value) are presented**** b)** tests are appropriate, there is p value, but reporting is incomplete *** c)** The statistical test is not appropriate or not described. |
| Evaluation | **a)** Very Good Studies: 9-10 stars  **b)** Good Studies: 7-8 stars  **c)** Satisfactory Studies: 5-6 stars  **d)** Unsatisfactory Studies: 0 to 4 stars |
